# Supplementary material for: Expanding the Library of 1,2,4-Oxadiazole Derivatives: Discovery of New Farnesoid X Receptor (FXR) Antagonists/Pregnane X Receptor (PXR) Agonists
Source: Molecules. 2023 Mar 21;28(6):2840. doi: 10.3390/molecules28062840 (PMC10057480; doi:10.3390/molecules28062840)
Supplement: Supplementary file 1 [file molecules-28-02840-s001.zip › molecules-2288978-supplementary.pdf]

Supplementary Materials

Expanding the library of 1,2,4-oxadiazole derivatives: discovery of new Farnesoid X Receptor (FXR) antagonists/Pregnane X Receptor (PXR) agonists

Claudia Finamore[1], Carmen Festa[1], Bianca Fiorillo[1,2], Francesco Saverio Di Leva[1], Rosalinda Roselli[3], Silvia Marchianò[3], Michele Biagioli[3], Lucio Spinelli[1], Stefano Fiorucci[3], Vittorio Limongelli[1,4], Angela Zampella[1], Simona De Marino[1*]

[1]Department of Pharmacy, University of Naples "Federico II", Via D. Montesano 49, 80131 Naples, Italy.

[2]Department of Pharmacological Sciences, Icahn School of Medicine at Mount Sinai, 1468 Madison Ave, 10029, New York, NY, United States.

[3]Department of Surgery and Biomedical Sciences, Nuova Facoltà di Medicina, Perugia, Italy.

[4] Faculty of Biomedical Sciences, Euler Institute, Università della Svizzera italiana (USI), Via G. Buffi 13, CH-6900 Lugano, Switzerland

Table of contents:

Figures S1 and S2. ^{1}H and ^{13}C-NMR spectra of compound **2** S2
Figures S3 and S4. ^{1}H and ^{13}C-NMR spectra of compound **3** S3
Figures S5 and S6. ^{1}H and ^{13}C-NMR spectra of compound **4** S4
Figures S7 and S8. ^{1}H and ^{13}C-NMR spectra of compound **5** S5
Figures S9 and S10. ^{1}H and ^{13}C-NMR spectra of compound **6** S6
Figures S11 and S12. ^{1}H and ^{13}C-NMR spectra of compound **7** S7
Figures S13 and S14. ^{1}H and ^{13}C-NMR spectra of compound **8** S8
Figures S15 and S16. ^{1}H and ^{13}C-NMR spectra of compound **9** S9
Figures S17 and S18. ^{1}H and ^{13}C-NMR spectra of compound **10** S10
Figures S19 and S20. ^{1}H and ^{13}C-NMR spectra of compound **11** S11
Figure S21. Transactivation assays on LXRα and LXRβ of compounds **5** and **11** S12

Figures S1-S2. ^{1}H (400 MHz) and ^{13}C NMR (100 MHz) in CD_3OD of compound **2**

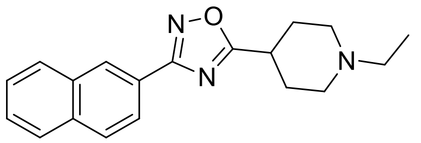

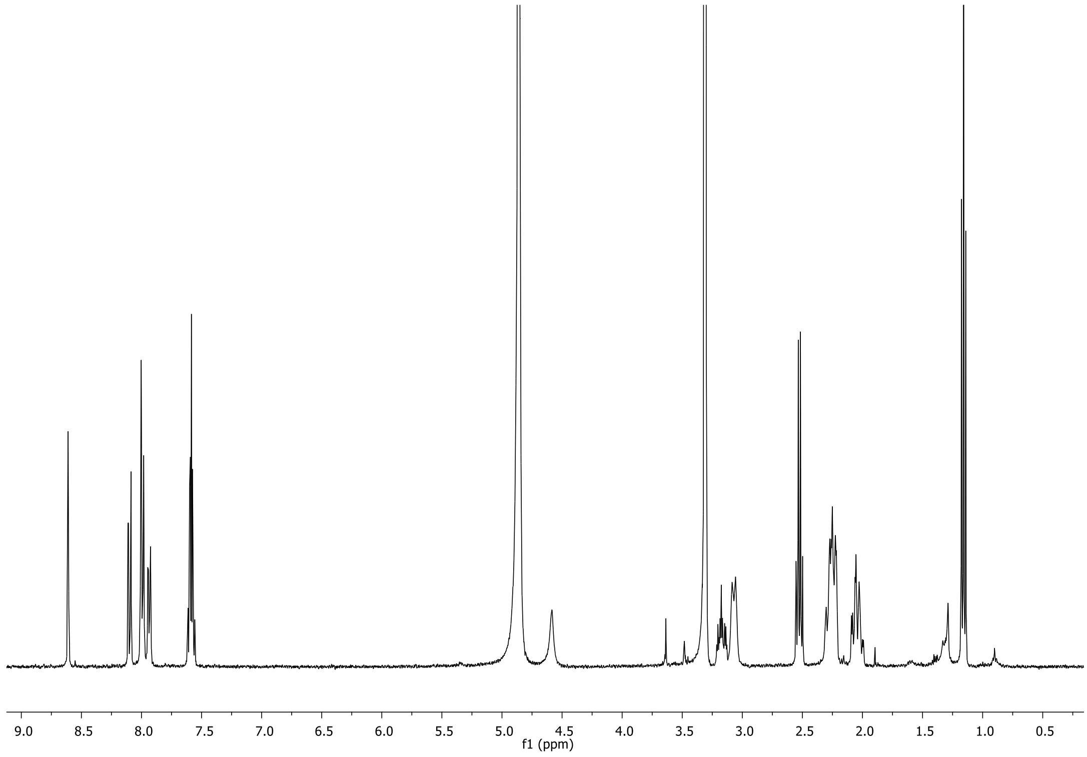

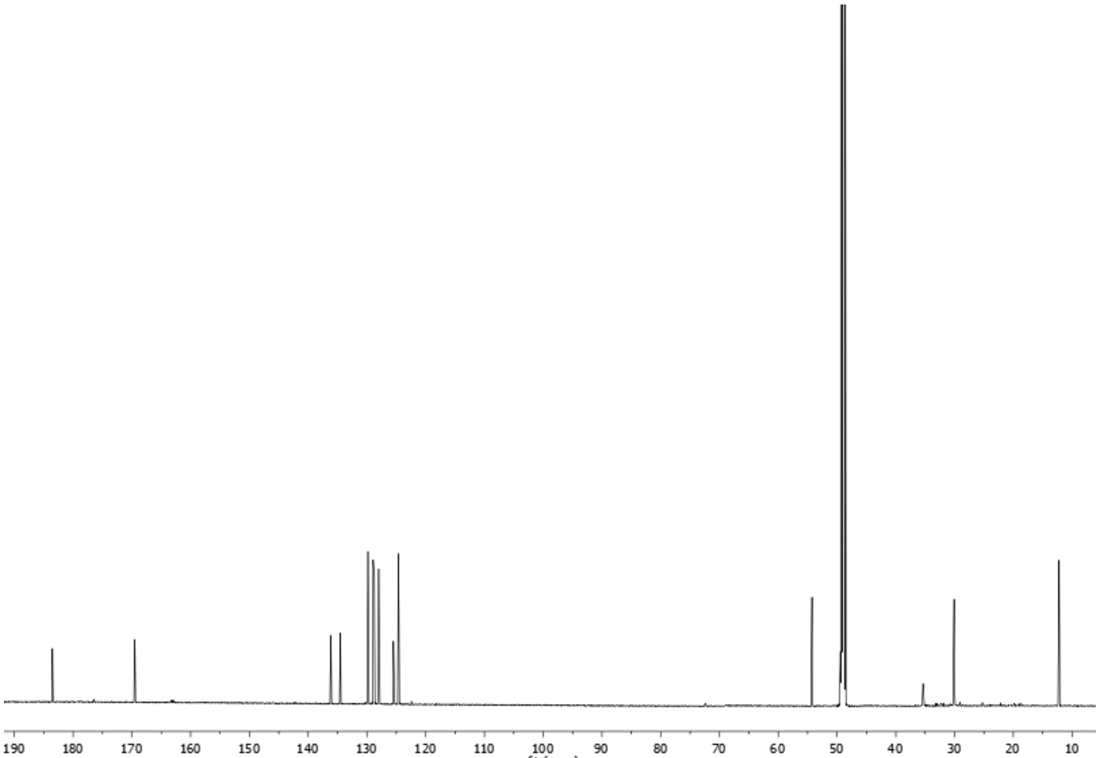

Figures S3-S4. ^{1}H (700 MHz) and ^{13}C (175 MHz) NMR in CD_3OD of compound **3**

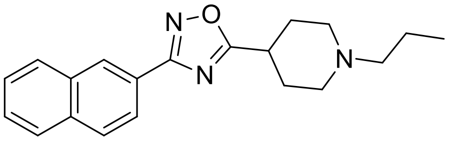

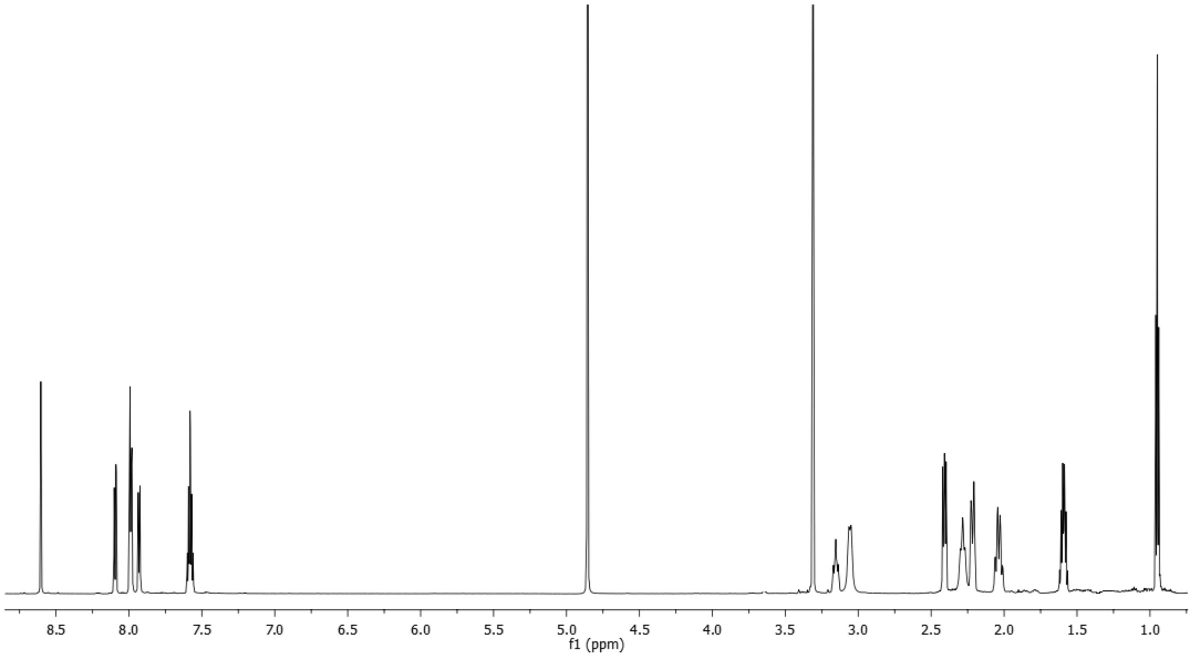

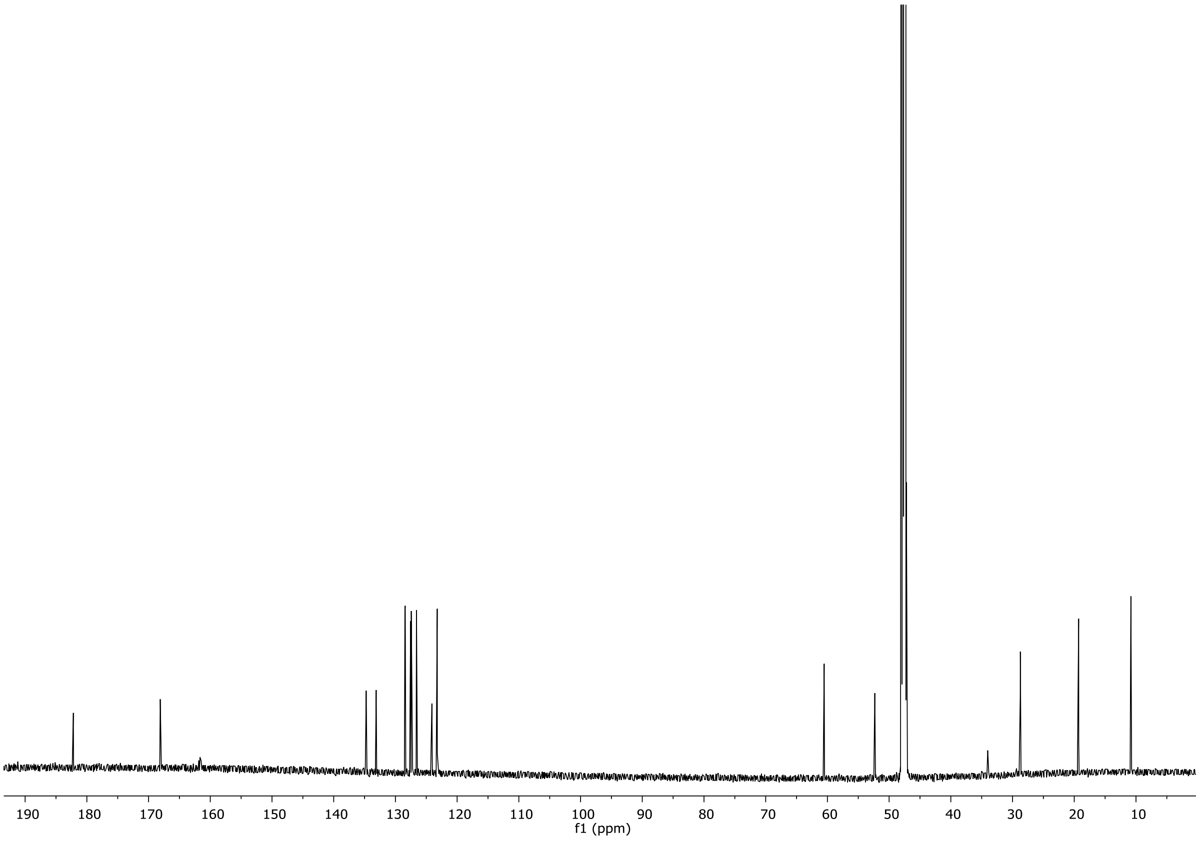

Figures S5-S6. ^{1}H (400 MHz) and ^{13}C NMR (100 MHz) in CD_3OD of compound **4**

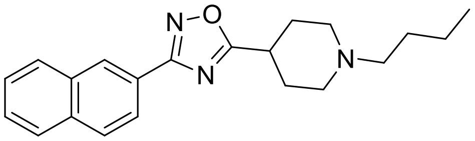

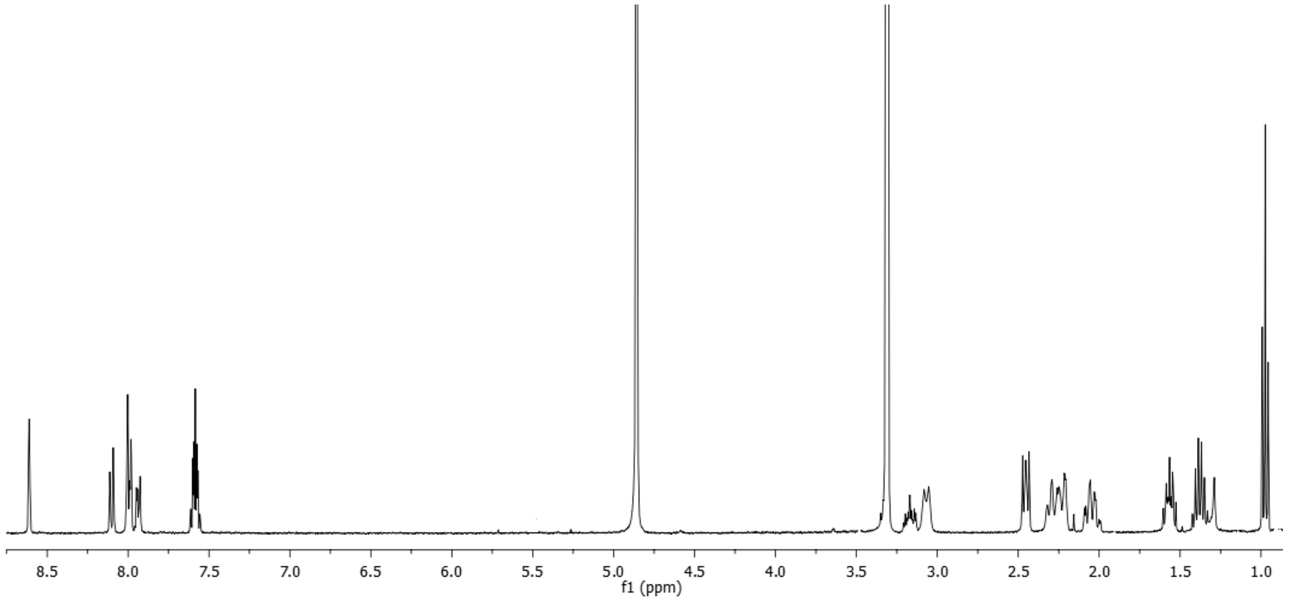

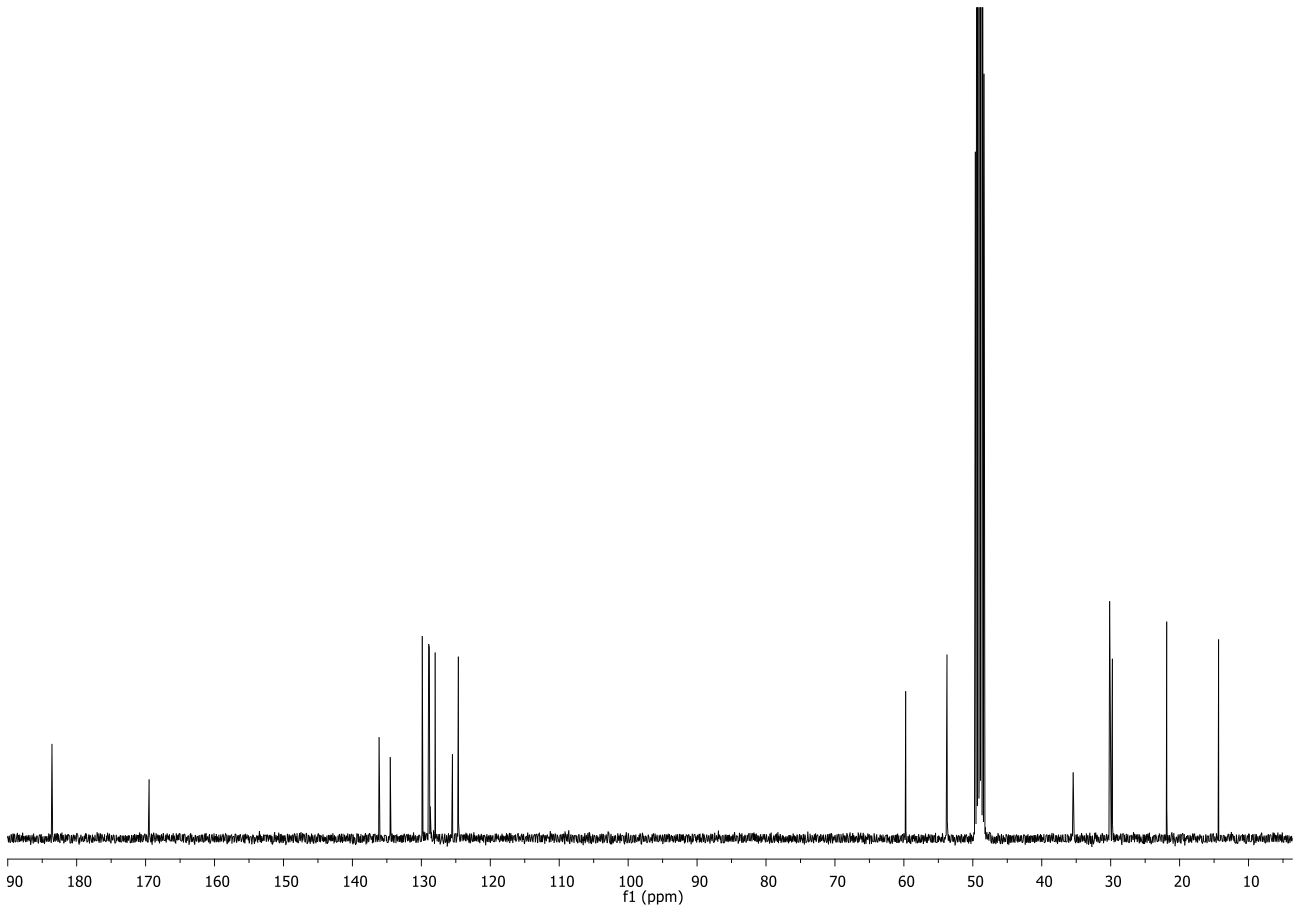

Figures S7-S8. ^{1}H (400 MHz) and ^{13}C NMR (100 MHz) in CD_3OD of compound **5**

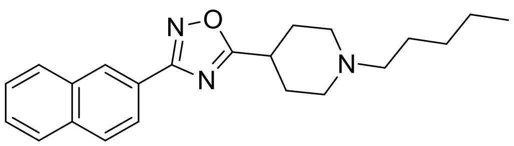

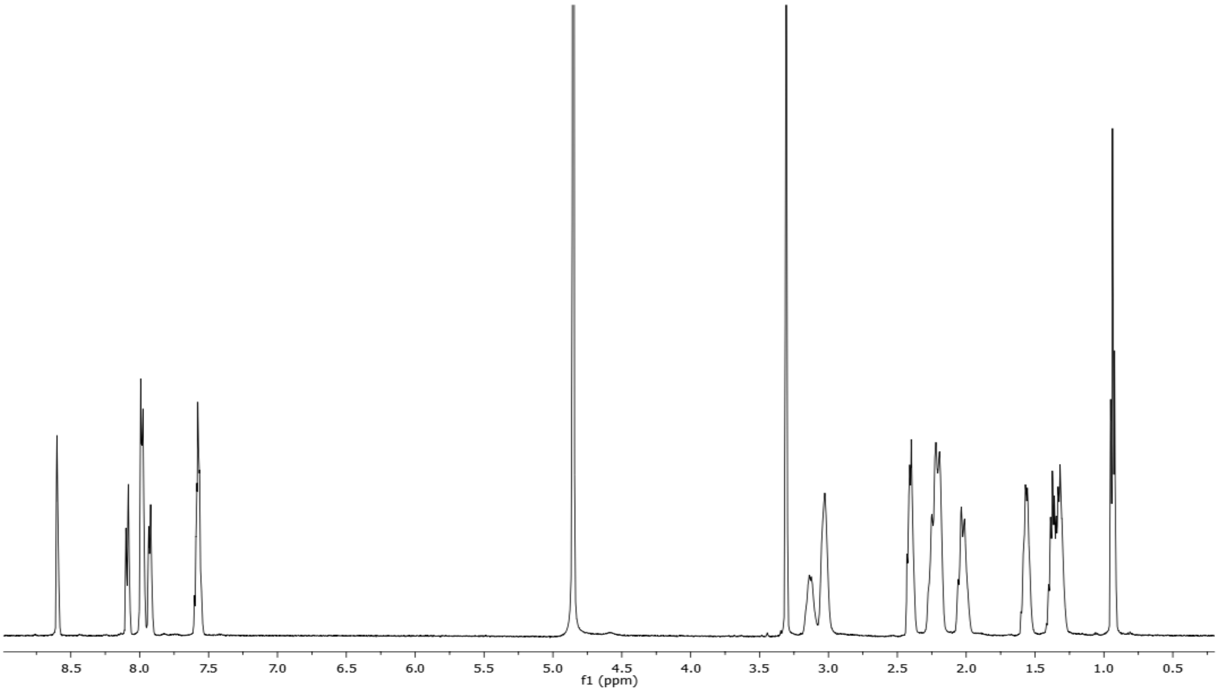

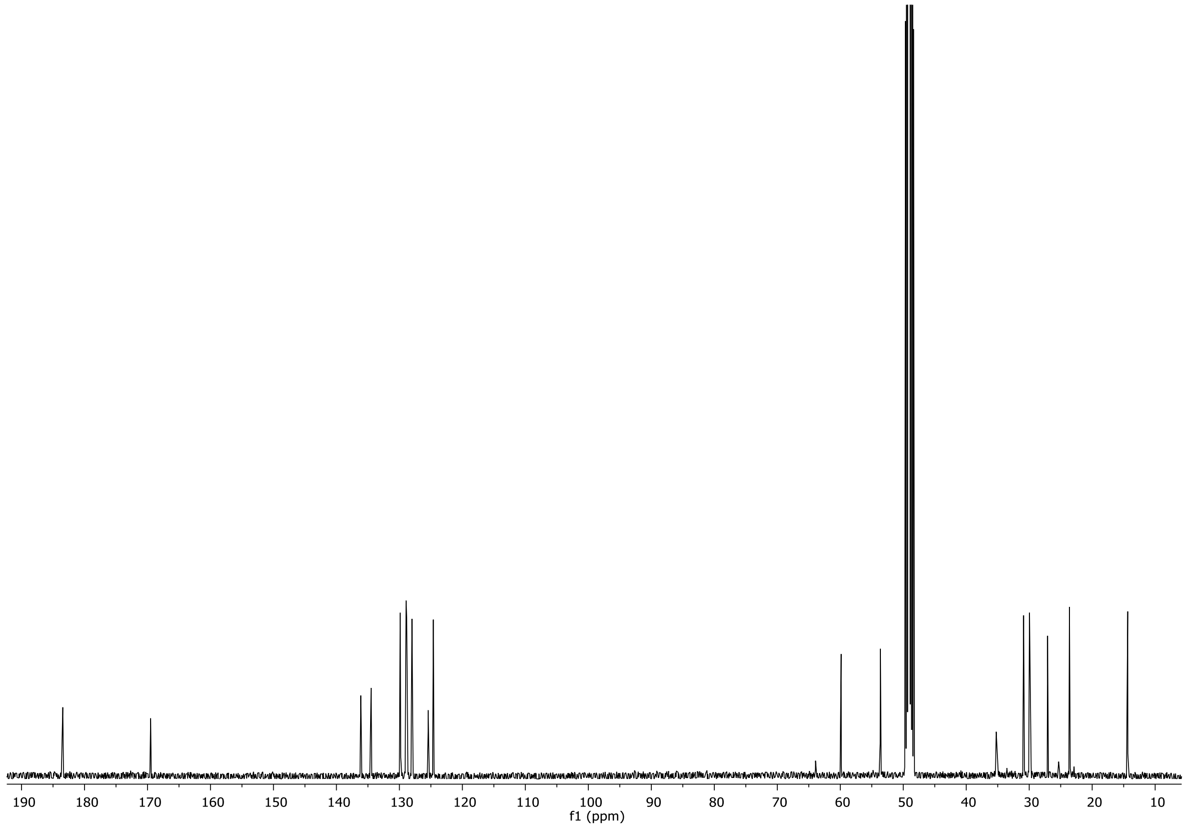

Figures S9-S10. ^{1}H (400 MHz) and ^{13}C NMR (100 MHz) in CD_3OD of compound **6**

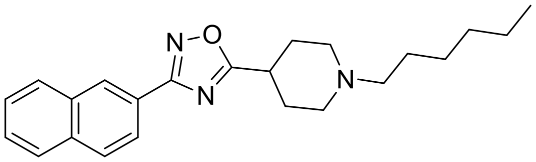

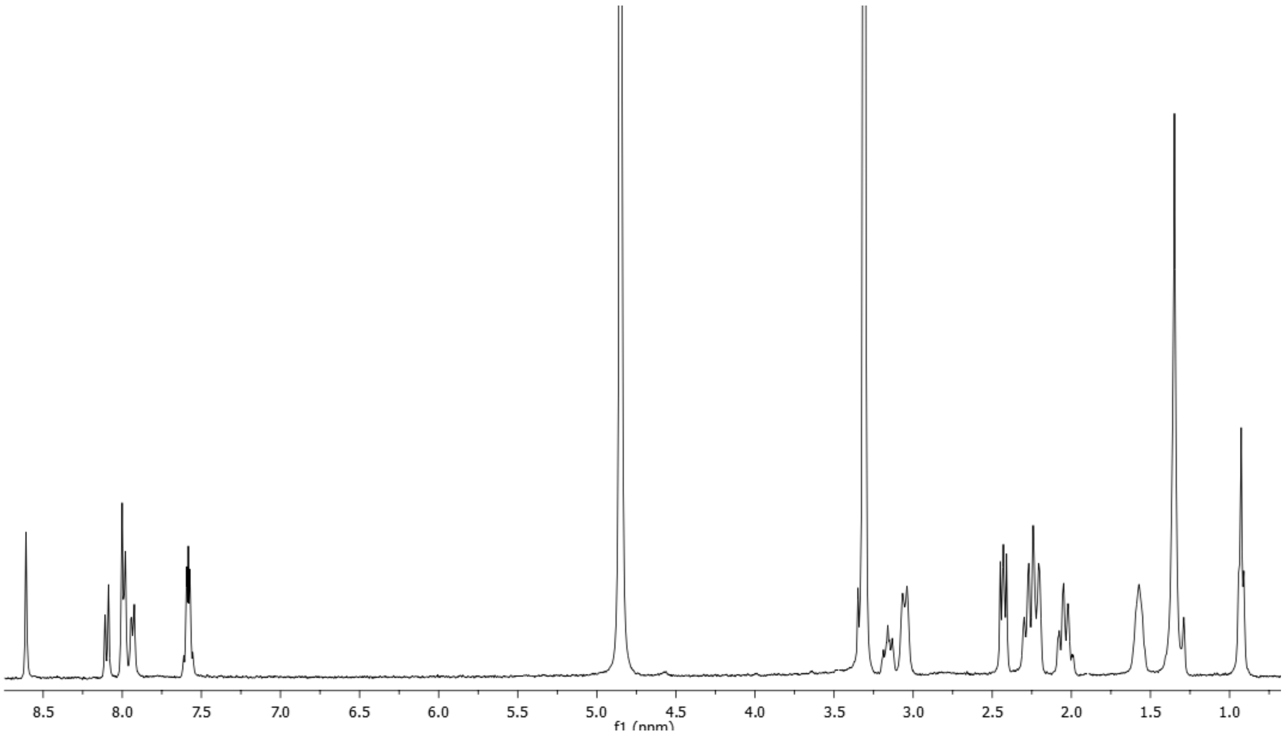

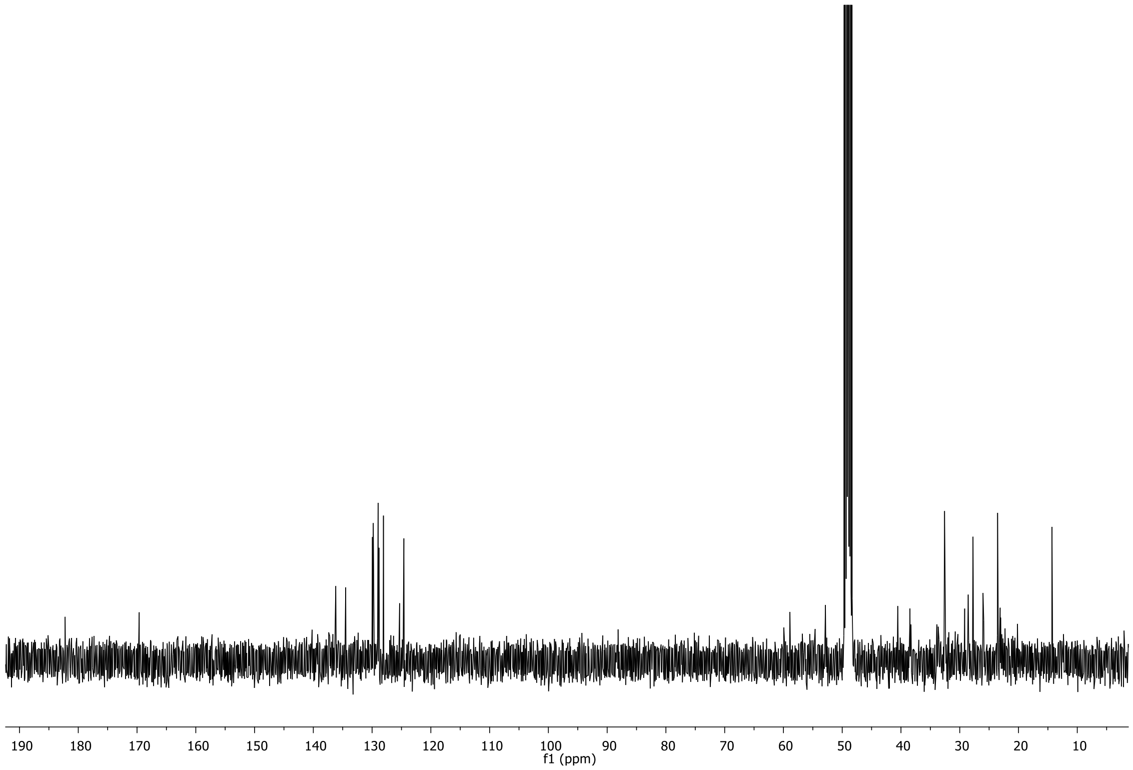

Figure S11-S12. ^{1}H (700 MHz) and ^{13}C NMR (175 MHz) in CD_3OD of compound **7**

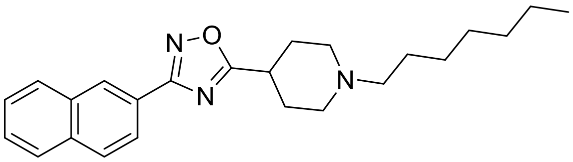

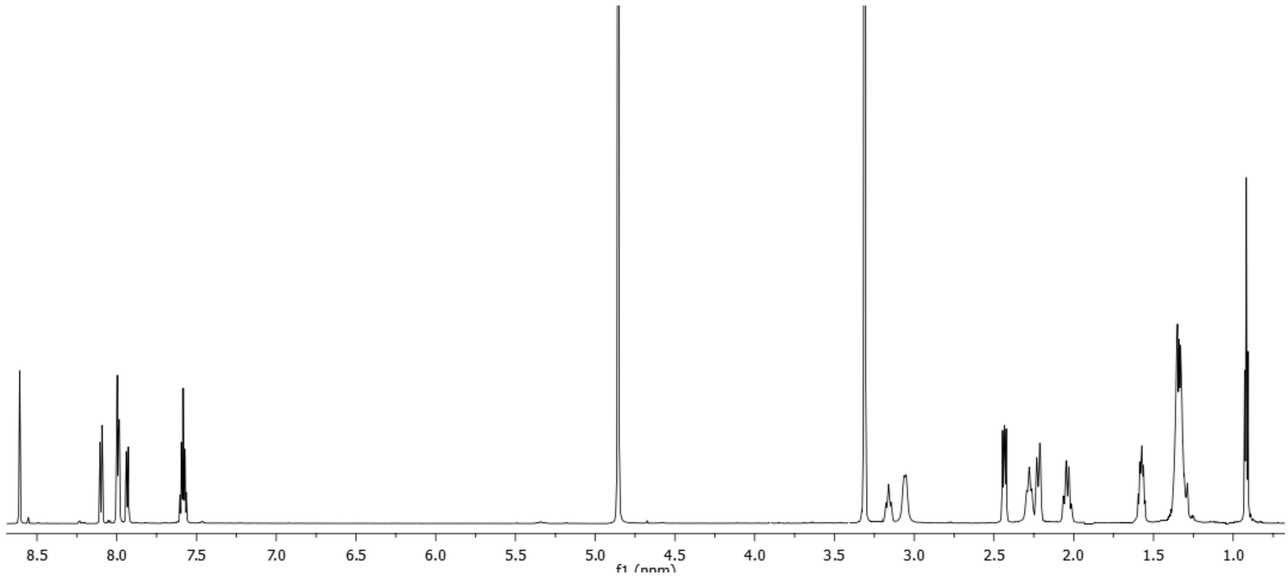

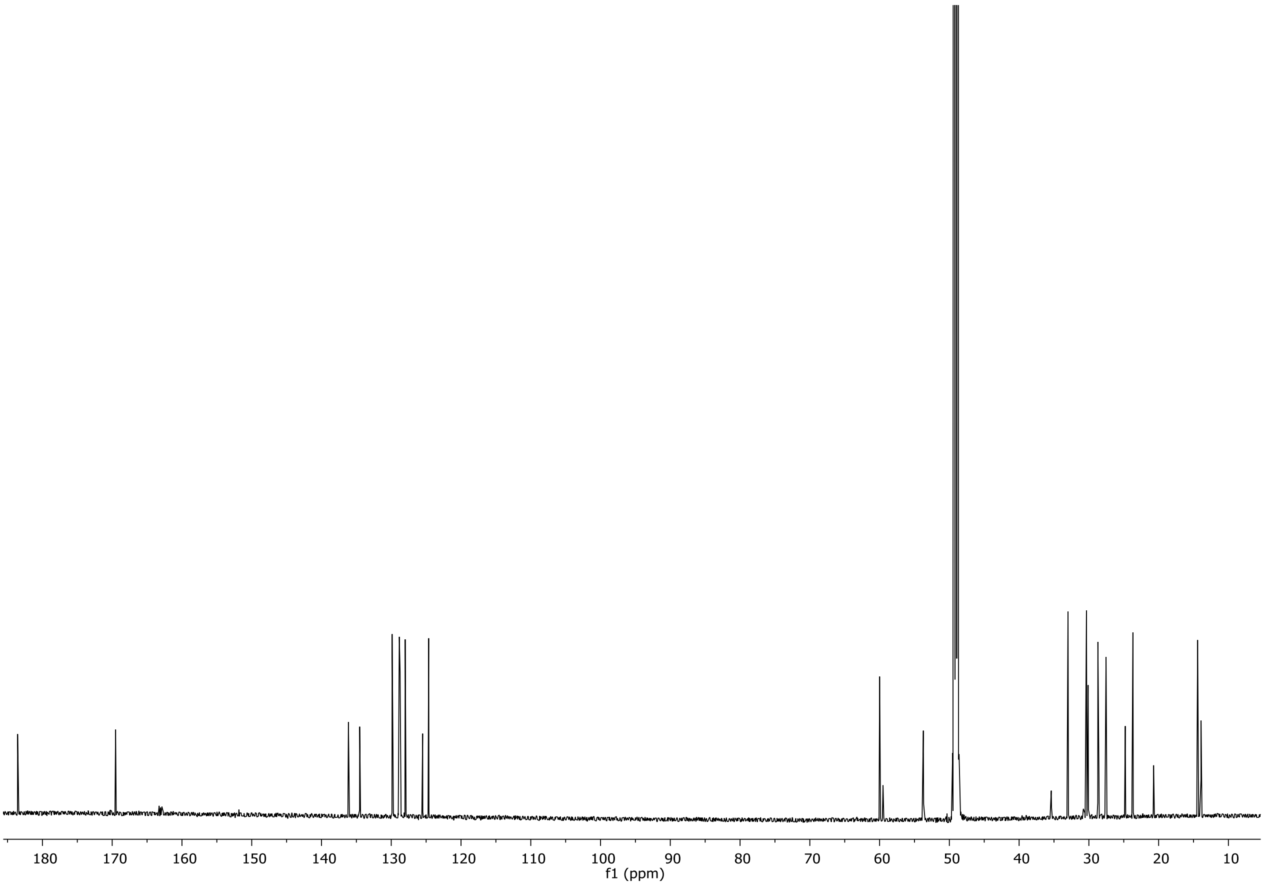

Figures S13-S14. ^{1}H (700 MHz) and ^{13}C NMR (175 MHz) in CD_3OD of compound **8**

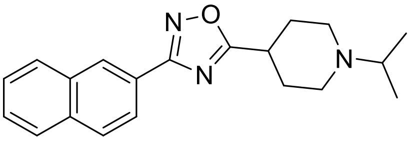

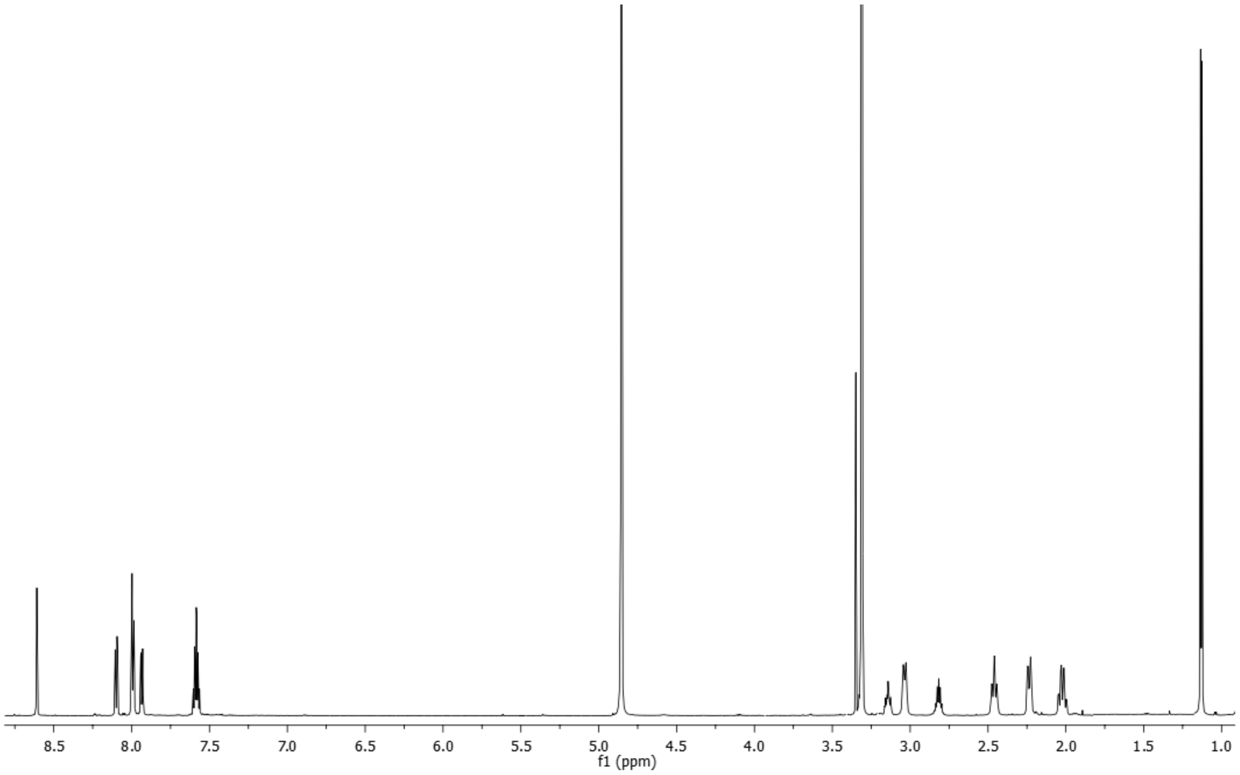

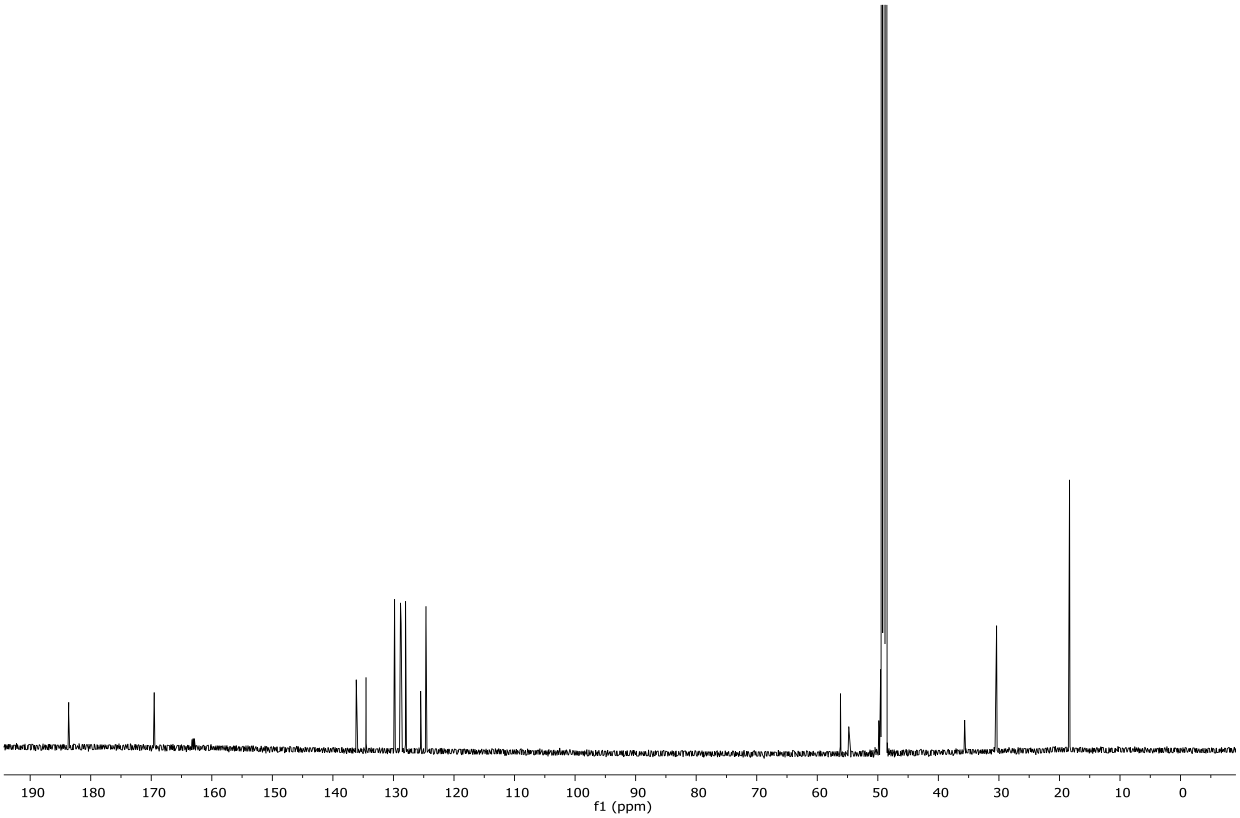

Figures S15-S16. ^{1}H (400 MHz) and ^{13}C NMR (100 MHz) in CD_3OD of compound **9**

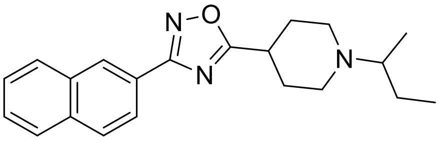

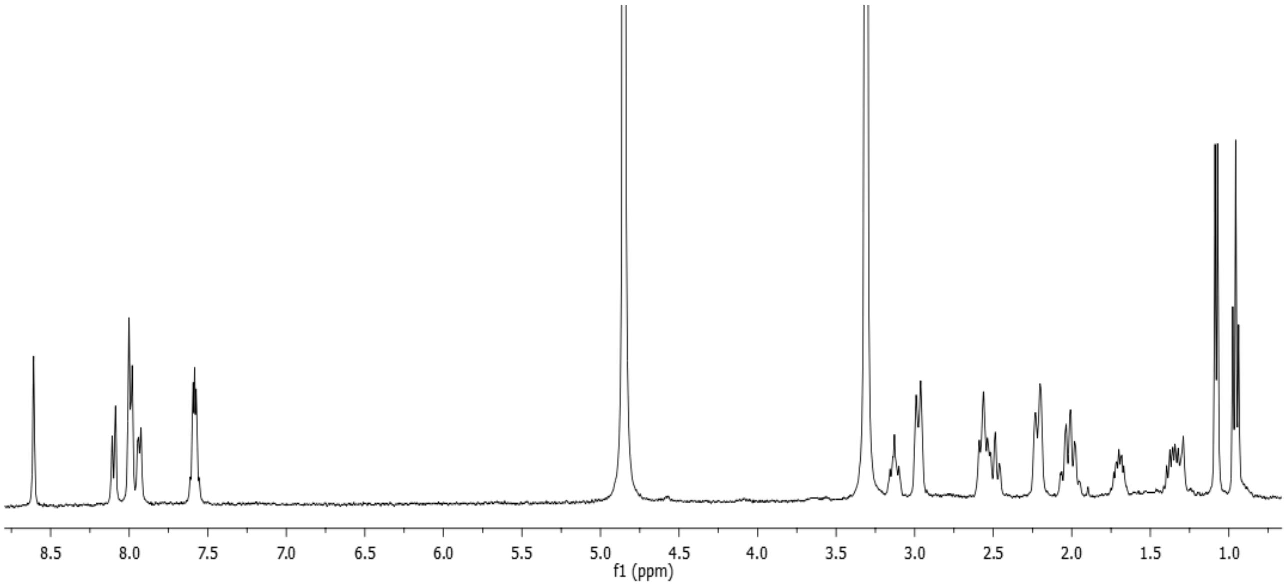

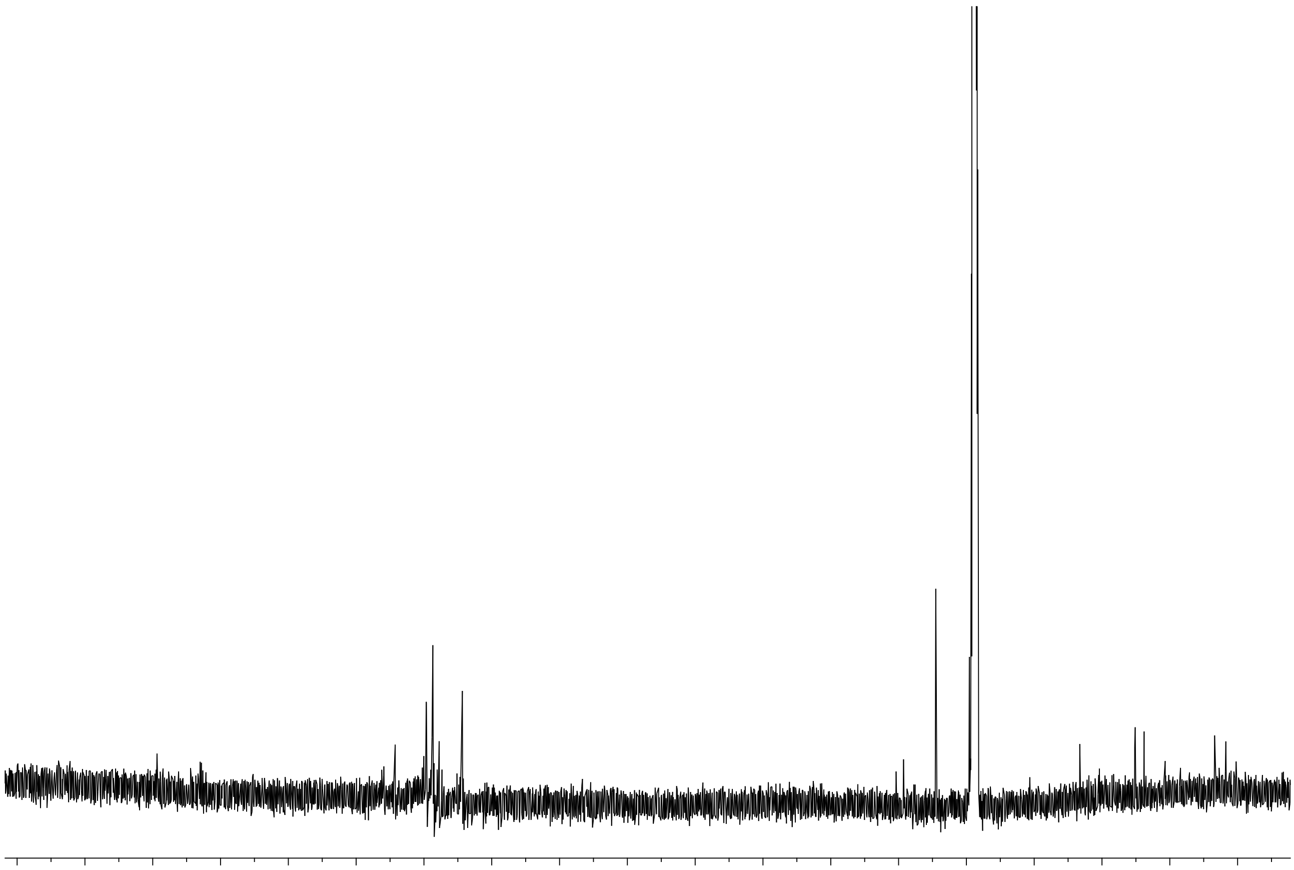

Figures S17-18. ^{1}H (400 MHz) and ^{13}C NMR (100 MHz) in CD_3OD of compound **10**

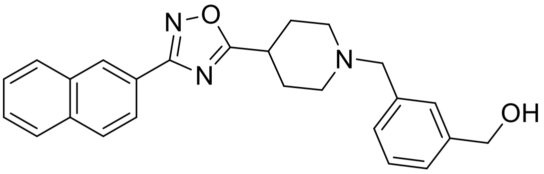

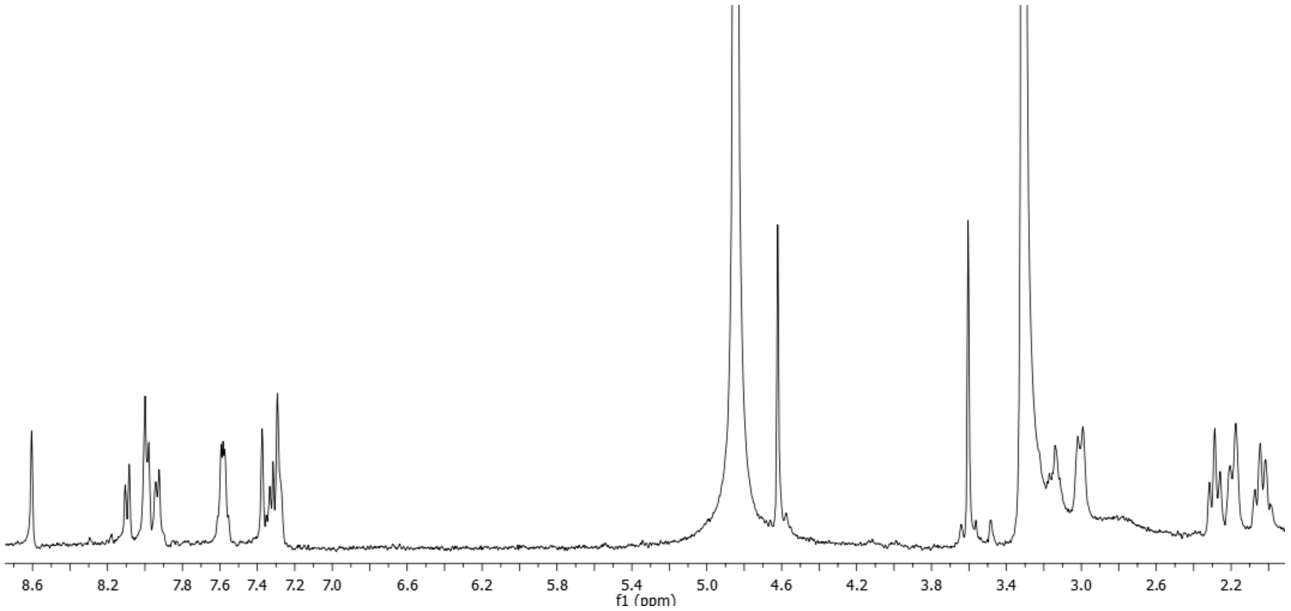

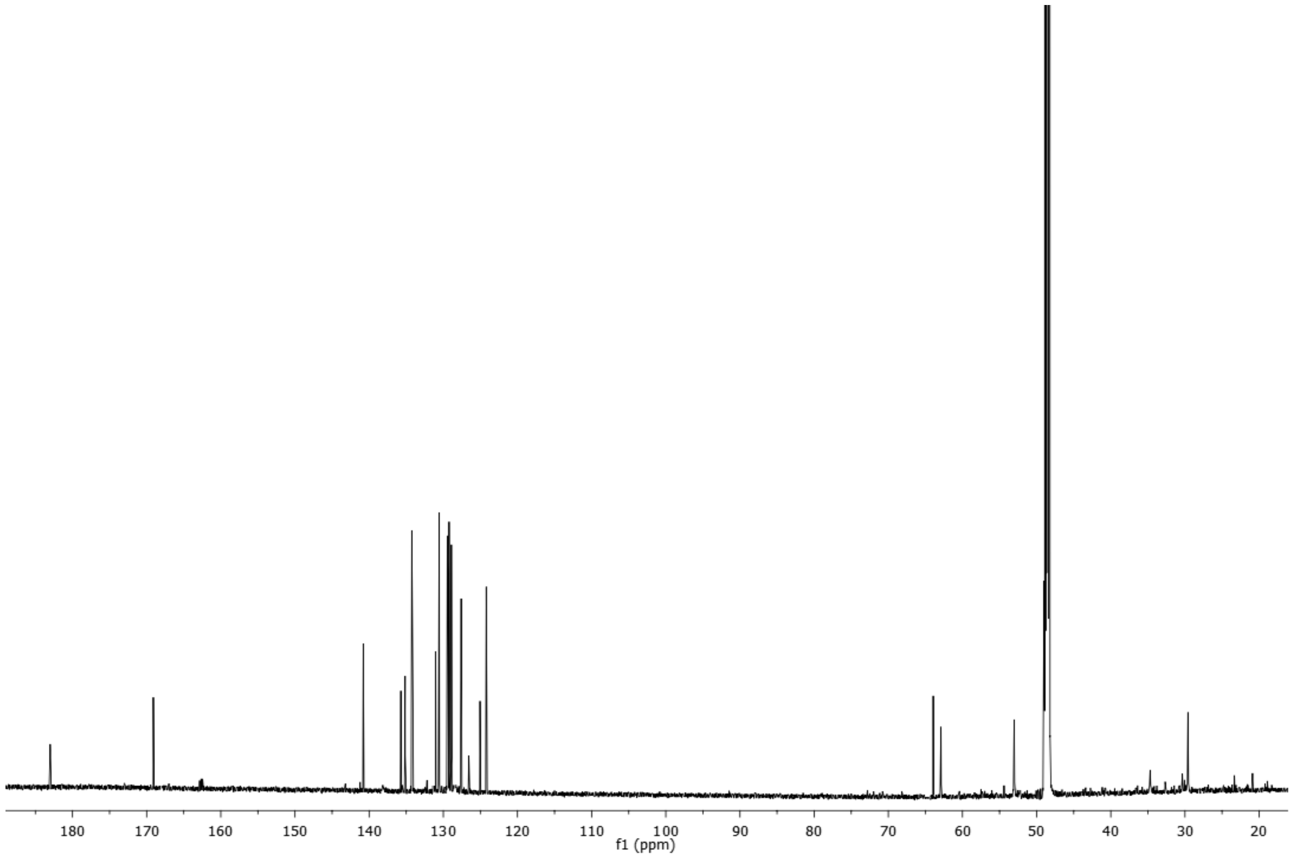

Figures S19-S20. ^{1}H (700 MHz) and ^{13}C NMR (175 MHz) in CD_3OD of compound **11**

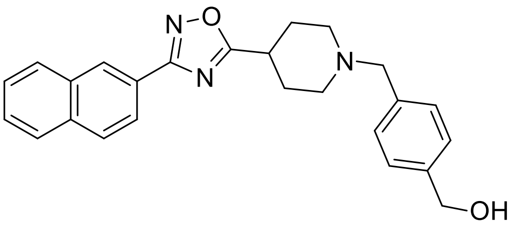

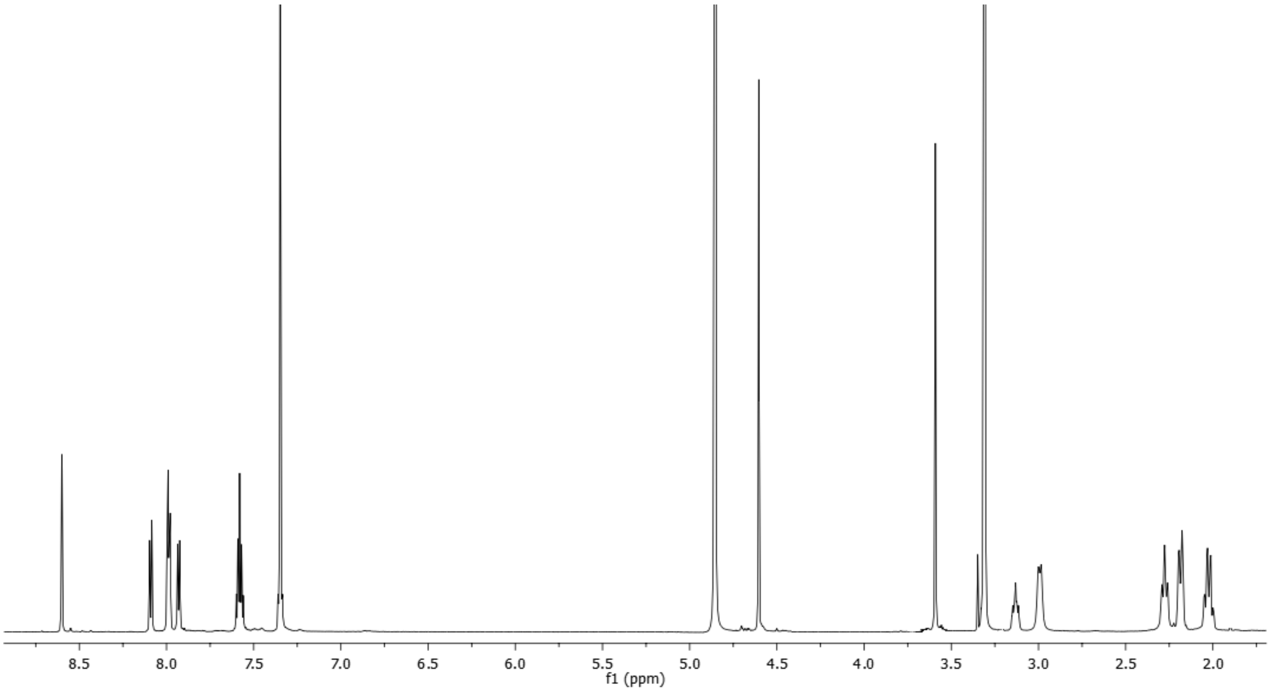

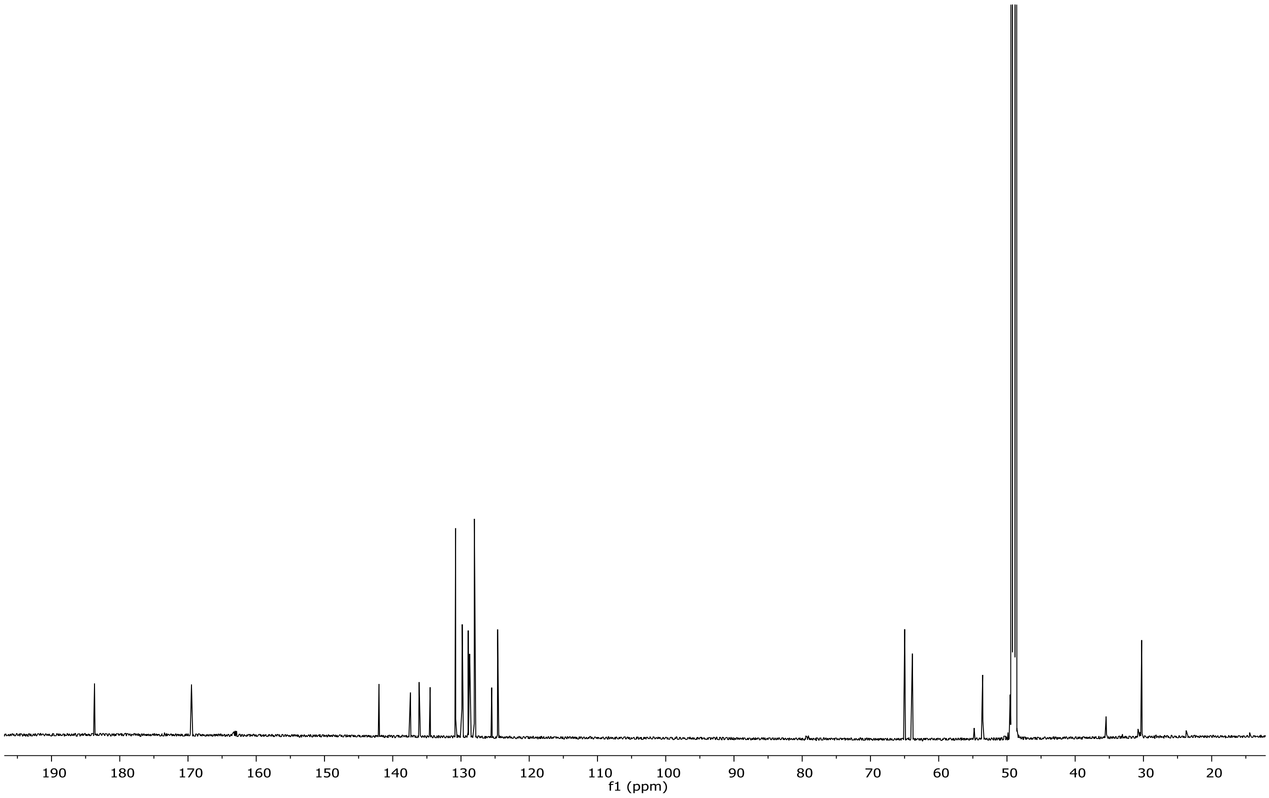

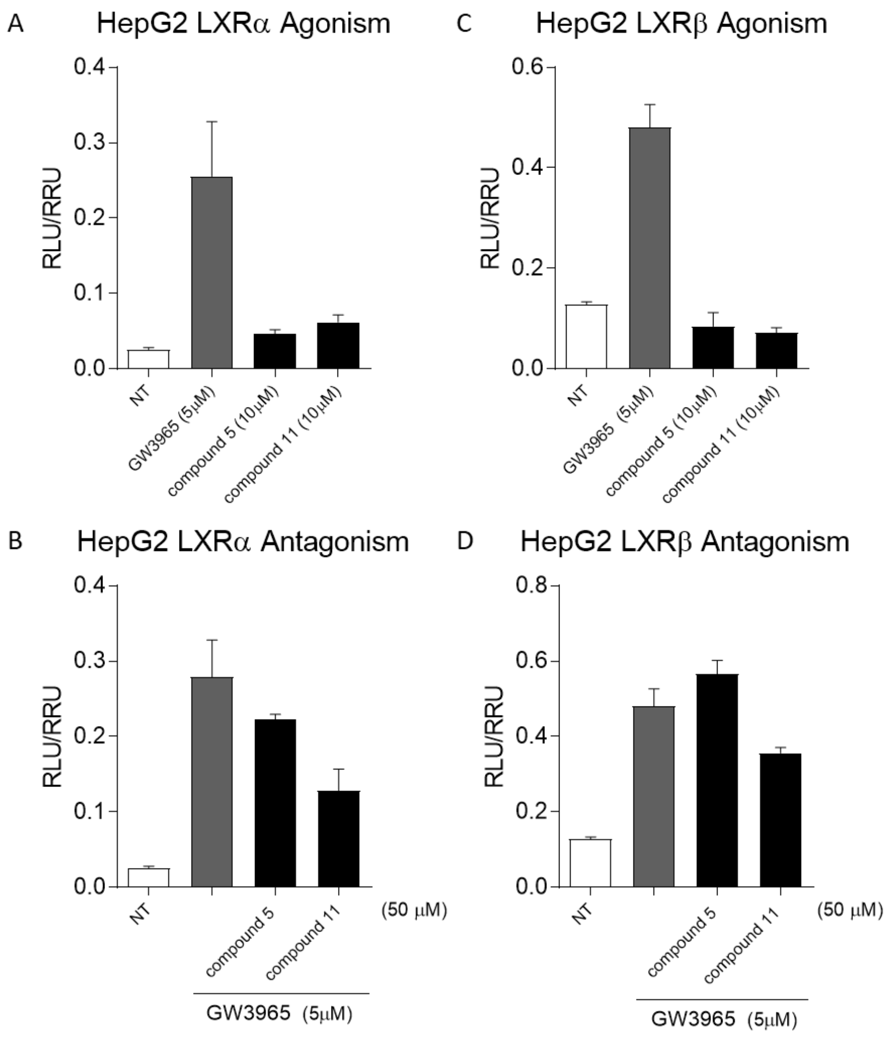

Figure S21. Activity towards LXRα and LXRβ in transactivation assay in agonism and antagonism. **A** and **C**) HepG2 cells were stimulated with **GW3965** (GW, 5 μM) as positive control and compounds **5** and **11** (10 μM); **B** and **D**) HepG2 cells were stimulated with 50 μM of compounds **5** and **11** in combination with the relative positive control. Results are expressed as mean ± SEM. *$p < 0.05$ versus not treated cells (NT).
